# Comparative plastomes sheds light on phylogeny of *Weigela*

**DOI:** 10.3389/fpls.2024.1487725

**Published:** 2024-10-29

**Authors:** Lei Wang, Fuxing Li, Kexin Zhao, Jie Yang, Haonan Sun, Xingyong Cui, Wenpan Dong, Enze Li, Ning Wang

**Affiliations:** ^1^ College of Horticulture and Plant Protection, Henan University of Science and Technology, Luoyang, China; ^2^ Laboratory of Systematic Evolution and Biogeography of Woody Plants, College of Ecology and Nature Conservation, Beijing Forestry University, Beijing, China; ^3^ College of Biology and Food, Shangqiu Normal University, Shangqiu, China; ^4^ College of Landscape and Tourism, Hebei Agricultural University, Baoding, China

**Keywords:** plastome phylogenetics, phylogeny, Weigela, Caprifoliaceae, maternal inheritance

## Abstract

*Weigela* Thunb. is a genus in the family Caprifoliaceae. All species in this genus have high ornamental and medicinal value. However, the genetic divergence between species and the phylogeny within *Weigela* is still unclear. Therefore, we sequenced and analyzed four plastomes from four different *Weigela* species to reveal the genetic divergence among species of this genus, and the phylogeny within *Weigela*. The four plastomes from *Weigela* ranged from 156,909 bp to 157,739 bp in size, and presented a typical circular quadripartite structure. Each complete plastome contained a pair of inverted repeat regions (23,592~24,957 bp), a larger single-copy (LSC) region (89,922~90,229 bp), and a small single-copy (SSC) region (17,668~20,429 bp). We identified three types of repeats, corresponding to 268 forward repeats, 128 palindromic repeats, and 867 tandem repeats, for a total of 1,263 long repeats. A total of 352 SSRs were identified from the four plastomes, and most of them were concentrated in the LSC region and the noncoding regions. Mononucleotide repeat units were the most frequently detected types of repeats, of which A/T repeat units were the most abundant. Three mutational hotspots (*trnH-psbA*, *trnR-ndhF*, and *trnN-ndhF*) were identified as candidate barcodes for *Weigela* species. *Weigela* belongs to Diervilloideae located at an early diverging position in the Caprifoliaceae. Within *Weigela*, *W. japonica* and *W. floribunda* were sister with *W. subsessilis* and *W. florida*. This study revealed the plastome structure and variation of four well-known *Weigela* species, and found three candidate barcodes for further study of four well-known *Weigela* species. In addition, the phylogenetic location of *Weigela* within the Caprifoliaceae was identified.

## Introduction

Given that the plastome is primarily characterized by maternal inheritance, it has been extensively employed in phylogenetic studies at different levels (even the population level) (Dong et al., 2021; Wang et al., 2022a, b; Zhang et al., 2007). Due to their stable structure, GC content, and non-recombination during the process of heredity, plastome sequences can often be used for species identification (Liu et al., 2023). Despite the highly conserved structure of the plastome, evolutionary events including gene or intron loss can also occur during the course of species evolution (Claude et al., 2022).

**Figure 1:**
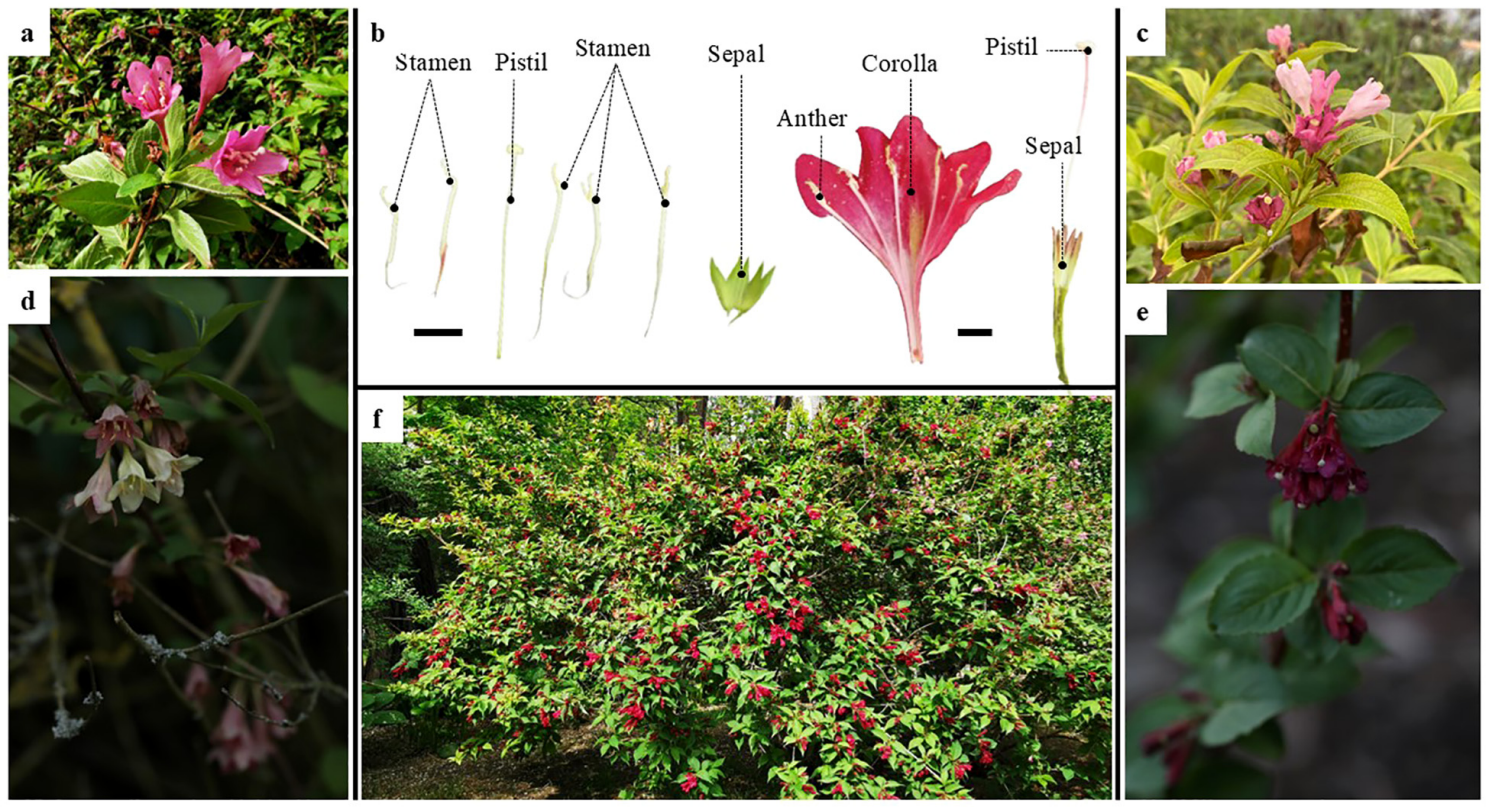
Floral anatomy of *Weigela florida* and its habitat. **(A)** Pink flowers of *Weigela florida*; **(B)** Floral anatomy of *Weigela florida*; **(C)** Pink flowers of *Weigela japonica*; **(D)** Flowers of *Weigela subsessilis*; **(E)** Flowers of *Weigela floribunda*; **(F)** Morphology and habitat of *Weigela florida*; Scale bars: 1.0 cm.

*Weigela* Thunb. belongs to Caprifoliaceae, and all species in this genus have high ornamental and medicinal value (Liang et al., 2013; Yamada and Maki, 2012). The genus contains about 11~12 species, having a discontinuous distribution pattern in America and Asia (Kim and Kim, 1999). The phylogeny based on the ITS showed that *Weigela* could be divided into three major clades, one core group and two single species clades (Kim and Kim, 1999). The flowers are large with bell-shaped or funnel-shaped corollas, solitary or in cymes of two to six flowers in the axils or tips of lateral branches, with white or pink-to-crimson petals (**Figure 1**). Because of their unique flower shape and multifarious colors, species of *Weigela* are a unique ornamental resource for urban greening. However, to date, there have been few studies on the phylogeny and genetic diversity of *Weigela*. Meeler combined morphological evidence with nuclear ITS and chloroplast gene (*trnS-G*) sequences to reconstruct the phylogeny of *Diervilla*, showing that *Diervilla* and *Weigela* are clustered in the Diervilloideae (Meeler, 2018), and that, because of the sister-group relationship between *Diervilla* and *W. middendorffiana* (Carrière) K.Koch, *Weigela* is not a monophyletic group (Kim and Kim, 1999; Meeler, 2018). In addition, studies on the phylogeny of the entire family (Caprifoliaceae) have rarely involved *Weigela*, despite the phylogeny within Caprifoliaceae having been studied extensively based on morphological characters (Donoghue et al., 2003; Sun et al., 2022; Donoghue et al., 2001) or molecular evidence (Jacobs et al., 2009; Wang et al., 2021, 2020; Li et al., 2024; Bell et al., 2001). So far, there has been no clear conclusion on the phylogenetic relationships of *Weigela*, which has seriously restricted the development and utilization of this genus, especially in the breeding of new, high-quality ornamental varieties.

Based on the abovementioned state of the research and the urgent problems still to be solved, our study aims to reveal the genetic diversity of *Weigela* using whole plastome sequences. Furthermore, in order to clarify the phylogenetic status of *Weigela*, this study aims to reconstruct the phylogeny of Caprifoliaceae using plastomic data. The main objectives of this study include the following three aspects: (1) To comprehensively compare and analyze the structural characteristics of plastomes between four *Weigela* species, (2) to reveal the genetic divergence within *Weigela*, (3) and to clarify the phylogeny of *Weigela* and verify the phylogenetic position of *Weigela* within the Caprifoliaceae.

## Materials and methods

### Plant material, DNA extraction, and genome sequencing

In this study, four species found mainly in northeast Asia of the core clade in *Weigela* were selected based on previous studies (Kim and Kim, 1999; Meeler, 2018). The tissue of four *Weigela* species were obtained from the National Botanical garden in Beijing, China. These samples were identified by Dr. Wenpan Dong from Beijing Forestry University, and voucher specimens of these samples were deposited in the Herbarium at the College of Horticulture and Plant Protection, Henan University of Science and Technology, Henan province, China. The fresh leaves were preserved in silica gel, and the detailed information of these samples is given in **Supplementary Table S1**. Total DNA was isolated from each sample using a modified CTAB method (Li et al., 2013) and detected through electrophoresis on 0.8% agarose gels. The library preparation and Illumina-based Nova PE150 sequencing were carried out at Novogene Biotechnology Co., Ltd. in Tianjin, China, resulting in a total of 6 Gb of raw data.

### Plastome assembly and annotation

The sequencing raw data were firstly filtered, and those low quality data located in the joints or ends were removed with Trimmomatic v0.39 (Bolger et al., 2014) using the default parameters. GetOrganelle v1.7.6.1 (Jin et al., 2020) was used to assemble the plastome with the default parameters. The plastomes were annotated by PGA (Plastid Genome Annotator) (Qu et al., 2019) with the reference of *W. florida* (Bunge) A. DC. (MN524626), and the annotation results were manually checked through Geneious Prime v2021 (Kearse et al., 2012) to ensure their accuracy, especially for those genes with high sequence differentiation and introns. Plastome maps of *Weigela* were drawn and visualized using OGDRAW v1.3.1 (Greiner et al., 2019). The annotated sequences of these complete plastomes were deposited in GenBank, with the accession numbers listed in **Supplementary Table S1**.

### Comparison of plastome structures

The comparative analyses of plastome structures and the sequence similarity between the four *Weigela* species were conducted by the program mVISTA (Mayor et al., 2000). The annotated plastome of *W. floribunda* (Siebold & Zucc.) K.Koch was used as a reference. The online program IRscope (Amiryousefi et al., 2018) was used to compare the variation in the junction regions between LSC, IRb, SSC, and IRa.

The REPuter program (Kurtz et al., 2001) and the Perl script MISA (Beier et al., 2017) were used to analyse the repeat sequences of these plastomes. Various types of long repeats, including forward, reverse, palindromic, and complementary were identified across the four *Weigela* plastomes by the REPuter online program, with a Hamming distance of 3 and a minimum repeat size of 30 bp. The Perl script MISA was used to identify sequence repeats, including six types of SSRs (mono-, di-, tri-, tetra-, penta-, and hexanucleotides). The minimum thresholds for each SSR type were set at 10, 5, 4, 3, 3, and 3, respectively.

### Divergence analyses

Firstly, we used MAFFT v7 (Katoh and Standley, 2013) to align the plastomes for identifing the mutational hotspot regions and genes. Subsequently, the nucleotide diversity (Pi) of the four plastomes were analysed by DnaSP v6.12.03 (Zhou et al., 2018), and the hotspot regions with high mutation rates were identified using a sliding window method. The step size was prescribed at 20 bp, and the window length was 100 bp.

### Phylogenetic inference

In addition to the four newly assembled plastomes of *Weigela*, we also collected all the published plastome sequences of Caprifoliaceae from NCBI (Benson et al., 2018). A total of 84 species were used for the phylogenetic analysis including four species of the subfamily Diervilloideae, 34 of the subfamily Caprifolioideae, five of the subfamily Dipsacoideae, eight of the subfamily Valerianoideae, three of the subfamily Morinoideae, 17 of the subfamily Linnaeoideae, nine of *Zabelia*, and four species of *Viburnum* and *Sambucus* as the outgroups. All the voucher information and GenBank accessions of each taxon are provided in **Supplementary Table S1**. A total of 83 genes, consisting of 79 protein-coding genes and four rRNA genes, were extracted from each plastome for phylogenetic analyses. All extracted sequences for each sample were aligned through MAFFT v7 (Katoh and Standley, 2013).

The phylogeny of the family Caprifoliaceae was inferred through ML (Maximum Likelihood) method and BI (Bayesian Inference) method. The ML tree of Caprifoliaceae was reconstructed by RAxML-NG (Kozlov et al., 2019) with the model of GTR+G, and the node support was evaluated by 1,000 bootstrap replicates. The BI tree was reconstructed using MrBayes v3.2 (Ronquist et al., 2012), and four Markov chains were run for 5,000,000 generations, and each 1,000 generations were used for tree sampling. The convergence was analyzed in Tracer v1.6 (Rambaut et al., 2018) for stationary distribution along with the effective size for each parameter. To ensure the stable state of each chain, the initial 25% of the trees were removed as burn-in, and the PP (posterior probabilities) were calculated. The remaining trees were utilized for building the BI tree with posterior probabilities.

## Results

### The general features of *Weigela* plastomes

With the Illumina sequencing platform, we produced an average of about 6Gb raw data for each sample. The coverage depth of these plastomes were estimated by mapping at more than 40×. The complete plastomes were 156,909~157,739 bp in length and presented a typical circular quadripartite structure. Each complete plastome comprised a pair of inverted repeat (IR) regions (23,592~24,957 bp), a larger single-copy (LSC) region (89,922~90,229 bp), and a small single-copy (SSC) region (17,668~20,429 bp). The circular maps of the four *Weigela* plastomes are shown in **Supplementary Figure S1**.

In total, 113 unique genes were annotated for each *Weigela* plastome, consisting of 79 protein-coding genes, 30 tRNAs, and four rRNAs. In addition, 14 genes including three protein-coding genes, four rRNAs, and seven tRNAs were duplicated within the IR regions. On account of the expansion of the IR region in some species of *Weigela* (*W. japonica* Thunb., *W. florida*, and *W. floribunda*), the *ycf1* within the IRb region evolved into a pseudogene (**Supplementary Figure S1**). Among these genes, 60 genes are related to self-replication, and 45 genes are involved in photosynthesis (**Supplementary Table S2**). Of all these genes identified, only *ycf3* contained two introns, whereas 10 protein-coding genes and six tRNA genes only contained one intron (**Supplementary Table S4**). The longest intron (2,558 bp), including the *matK* gene, are located in the *trnK*-UUU gene. The *rps12* gene is a trans-spliced gene, with its 3’ end situated in the IR region and its 5’ end in the LSC region. The total GC content of these complete plastomes was 37.9%~38.0%, and the GC content of the IR region (42.8%~43.3%) exceeded that of the LSC region (36.3%~36.4%) and the SSC region (32.7%~33.1%).

### Repeat sequences and SSR analysis

**Figure 2:**
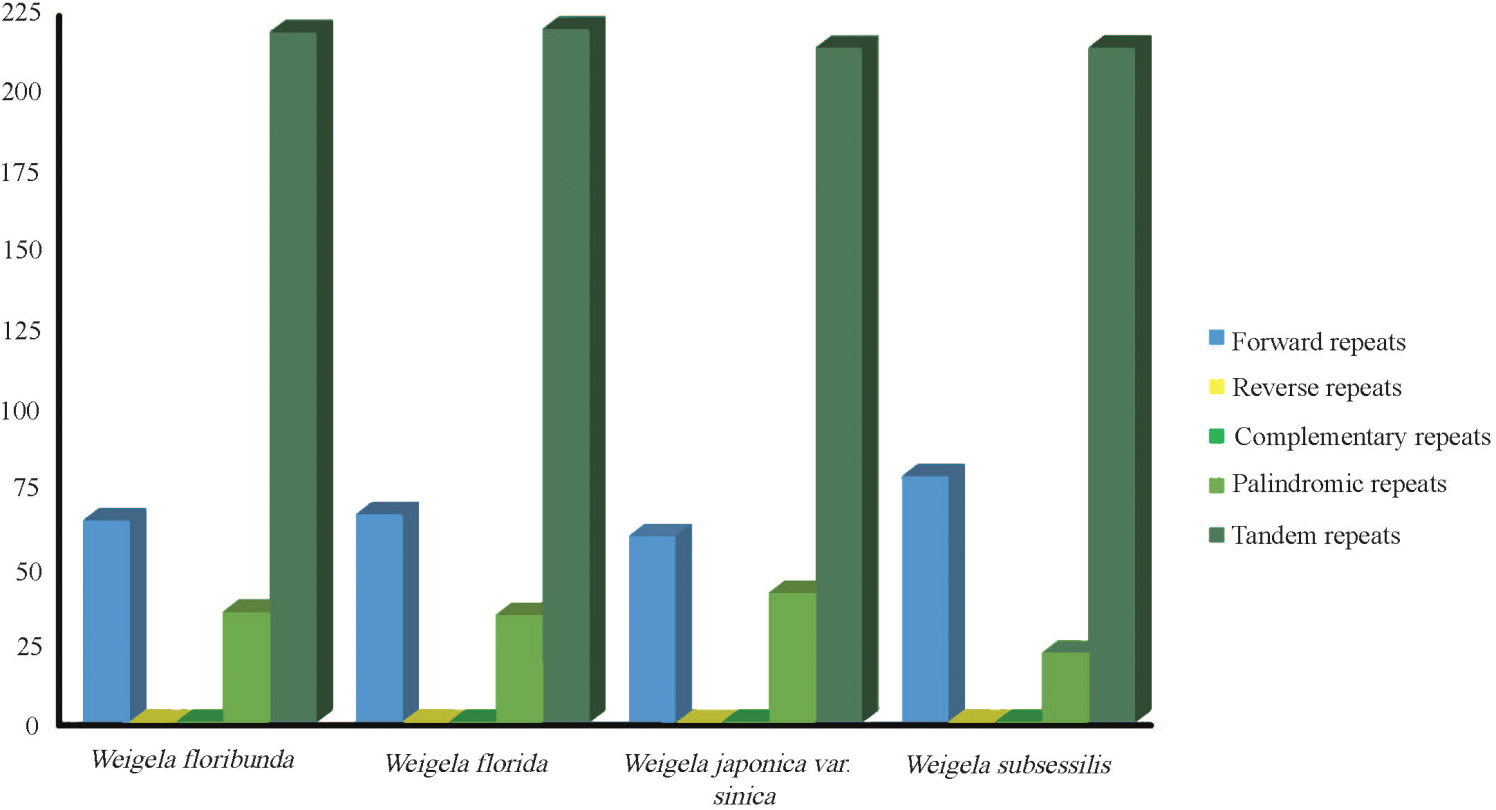
Analysis of repeated sequences in the plastomes of four *Weigela* species. Types and numbers of interspersed repeats within the four plastomes.

The repeat sequences of these four complete plastomes were investigated. The long repeat sequences greater than 30 bp were selected for analysis. From the four plastomes, we identified three types of repeats. There were 268 forward repeats, 128 palindromic repeats, and 867 tandem repeats, with a total of 1,263 long repeats. The number of tandem repeats was the highest among these, corresponding to 219 (*W. floribunda*), 220 (*W. florida*), and 214 (*W. japonica* var. *sinica* and *W. subsessilis* (Nakai) L.H.Bailey), while the number of palindromic repeats was the lowest, at 34 (*W. floribunda*), 33 (*W. florida*), 40 (*W. japonica* var. *sinica*), and 21 (*W. subsessilis*). Reverse and complementary repeats were not detected (**Figure 2**). Most of the long repeat sequences were mainly located in the noncoding areas, covering intergenic regions and intron regions. Several of the long repeat sequences were present in the shared genes, for instance *accD*, *trnN*, and *ycf2* (**Supplementary Table S3**).

**Figure 3:**
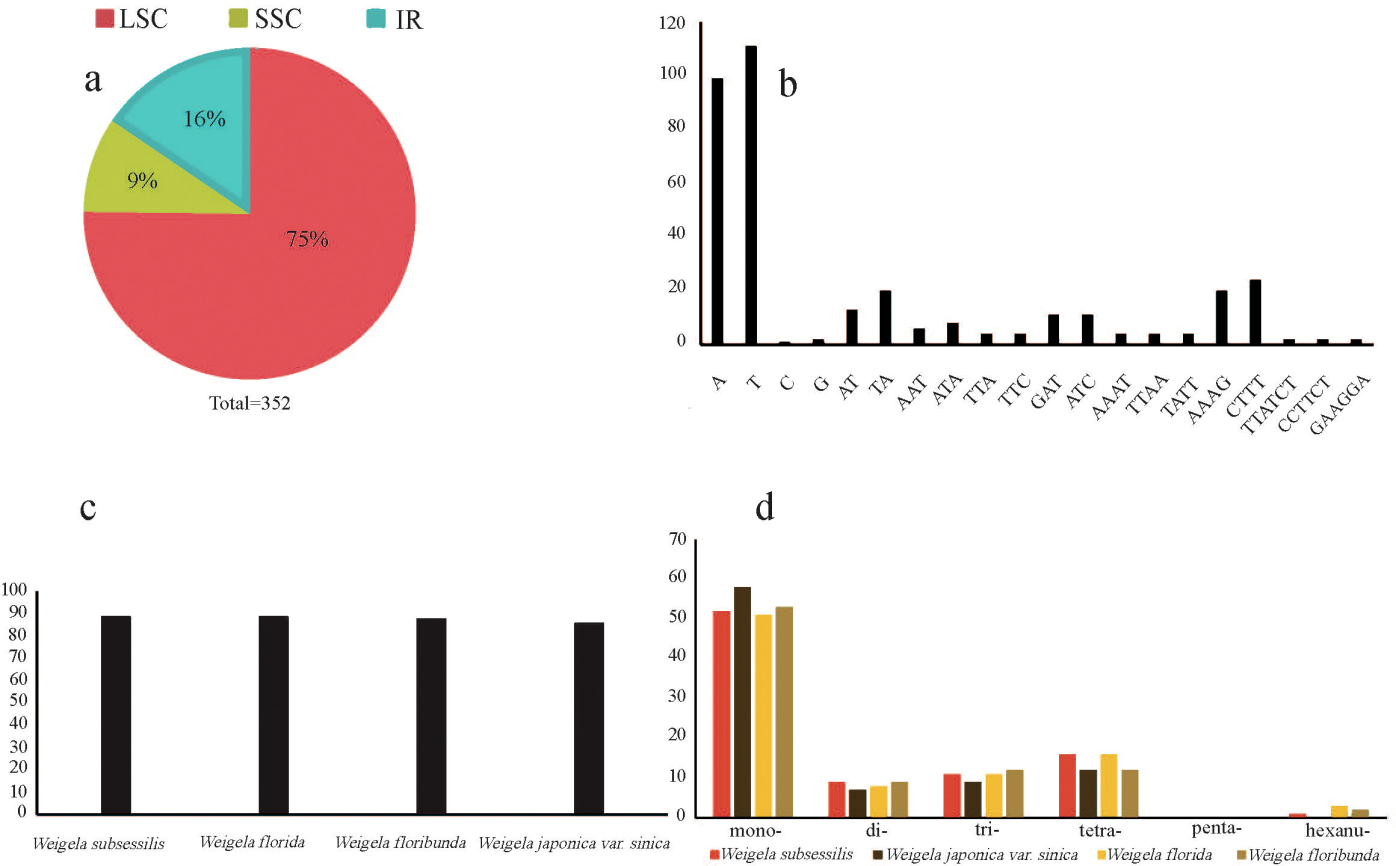
Frequency of the simple sequence repeat (SSR) sequences in the four *Weigela* plastomes. **(A)** The proportion of SSRs within the LSC, SSC, and IR regions; **(B)** Frequency of SSRs with the nucleotide motifs from di- to penta-; **(C)** The number of SSRs identified in the four *Weigela* species; **(D)** Number and type of SSRs.

A total of 352 SSRs were found in these four plastomes, with the number of SSRs per species being 89 (*W. subsessilis* and *W. florida*), 88 (*W. floribunda*), and 86 (*W. japonica* var. *sinica*). Most of the SSRs were concentrated in the LSC region, accounting for 75% of the total, while the number of SSRs in the IR and SSC regions was relatively small, accounting for 16% and 9% of the total, respectively (**Figures 3A, C**). The most common types of repeats detected were mononucleotide repeat units, of which the A/T repeat units were the most common. Among the dinucleotide and tetranucleotide repeat units, AT/TA and AAAG/CTTT were the most frequent units respectively. Pentanucleotide repeats were absent from these four plastomes of *Weigela*, whereas a small number of repeats were detected in the hexanucleotide repeats, except for *W. japonica* var. *sinica* (**Figures 3B, D**). We also found several SSRs within the protein-coding gene regions, including *rpoC1*, *trnG*, and *atpF* (**Supplementary Table S4**).

### Analysis of the plastome structure

Our comparative analysis of the *Weigela* plastomes showed that all four species have consistent gene content and very similar genome structures (**Table 1**, **Supplementary Table S2**, **Supplementary Figure S1**). All four plastomes showed a typical circular quadripartite structure, and the total genome size ranged from 156,909 bp (*Weigela japonica* var. *sinica*) to 157,739 bp (*Weigela florida*). The size of the LSC ranged from 89,922bp to 90,229 bp, and the size of the SSC was 17,668~20,429 bp. The four *Weigela* plastomes have consistent gene content and very similar GC content (37.9~38.0%) but differed by region. The GC content within the IR regions (42.8~43.3%) exceeded that of the LSC (36.3~36.4%) and SSC (32.7~33.1%) regions.

**Table 1:** Basic features of the plastome from the four species of *Weigela*.

Species		*W. subsessilis*	*W. japonica var. sinica *	*W. florida*	*W. floribunda*
Accession number		ENC851158	ENC851161	ENC851167	ENC851171
Length (bp)	Total	157,840	156,909	157,739	157,146
	LSC	90,229	89,922	90,023	90,058
	SSC	20,429	17,677	17,804	17,668
	IR	23,592	24,656	24,957	24,711
GC content (%)	Total	38.0	38.0	38.0	37.9
	LSC	36.4	36.3	36.4	36.3
	IR	43.3	42.8	42.9	42.8
	SSC	33.1	32.9	32.7	32.9
Gene numbers	Total	128	128	128	128
	Protein-coding gene	83	83	83	83
	tRNA gene	37	37	37	37
	rRNA gene	8	8	8	8

**Figure 4:**
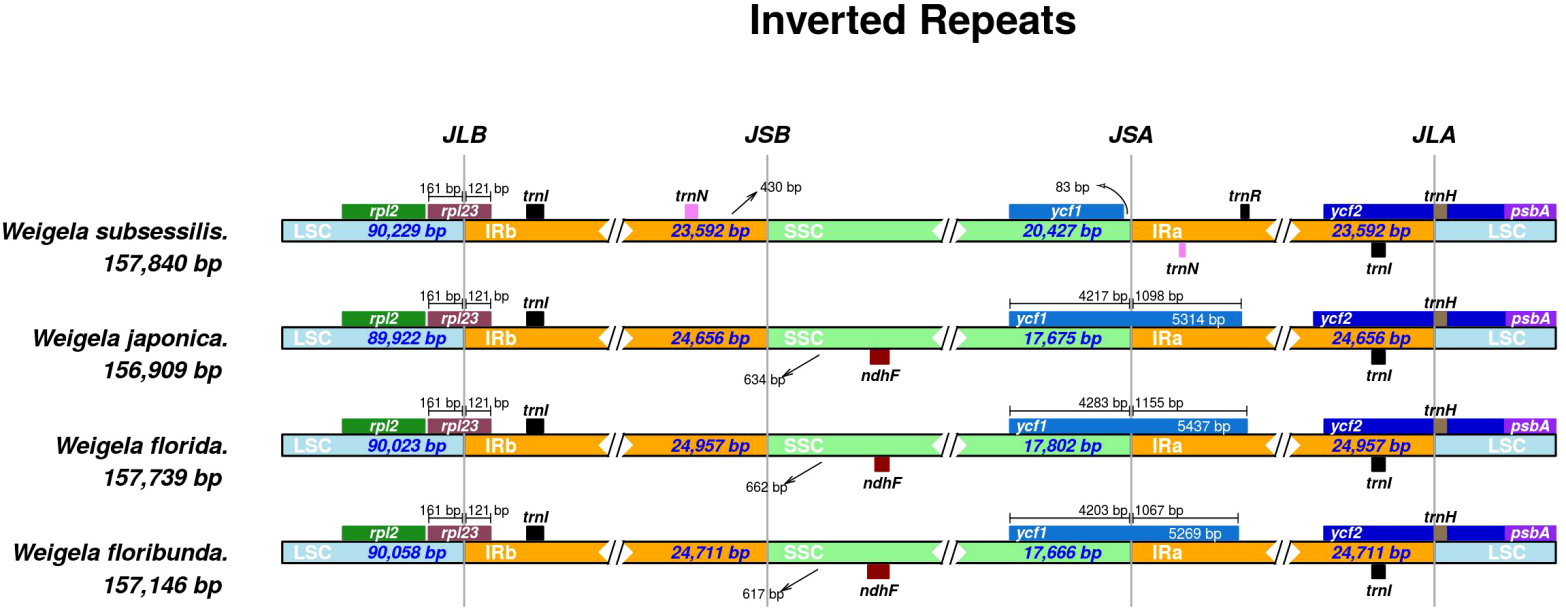
Comparison of the boundaries between LSC, SSC, and IR regions across the four *Weigela* species. The distance in the figure is not to scale.

Multiple sequence alignments indicated that there were no significant genomic rearrangements or large inversions in these four *Weigela* plastomes (**Figure 4**). These four plastomes of *Weigela* showed high conservation not only in gene order but also in basic features. As expected, the coding regions and inverted repeat (IR) regions were more conserved than the noncoding regions and single-copy (SC) regions. The intergenic spacer regions exhibited greater variability, such as *trnH-psbA*, *trnR-ndhF*, and *trnN-ndhF*. Moreover, only *ycf1* displayed a high level of variation across all the coding regions.

Multiple sequence alignments from mVISTA indicated that the IR regions of the four *Weigela* plastomes were rather conserved (**Table 2**), and structural variation was only observed in the boundaries between SSC and IR regions (**Figure 4**). Among these four plastomes, there were two types of SSC/IR boundary identified. *W. subsessilis* belonged to a type in which *ycf1* was located only in the SSC region, and 83 base pairs were identified between the *ycf1* and IRa boundary in this species. The other three species displayed a different type of structure with the *ycf1* gene spanning the boundaries between SSC and IRa regions. This result showed that the expansion of the IR regions caused a duplication of *ycf1* in the plastomes of *W. japonica*, *W. florida*, and *W. floribunda*, by 1,089, 1,155, and 1,067 bp, respectively. Moreover, an intergenic region of 430 bp was found between the *trnN* and the boundaries the LSC/IRb in *W. subsessilis*. Hundreds of base pairs were identified between the *ndhF* and IRb boundary in three species, namely, *W. japonica*, *W. florida*, and *W. floribunda*, by 634, 662, and 617 bp, respectively. These results indicated that there were dynamic variations in the boundaries between the SSC/IRa and the IRb/SSC among these four species. The *rpl23* gene crossed the LSC/IRb boundary in all the four plastomes with the length of 121 bp in the IRb region. The *trnl* and *trnH* genes were situated at the LSC/IRa boundary.

**Table 2:** Analyses of variable sites in plastomes from *Weigela*.

	Number of sites	Number of variable sites	Number of informative sites	Nucleotide diversity
LSC region	90,058	377	152	0.00232
SSC region	17,668	196	47	0.00554
IR region	24,711	180	50	0.00291
Compiete cp genome	159,867	919	302	0.00288

### Plastome sequence divergence and barcode identification

**Figure 5:**
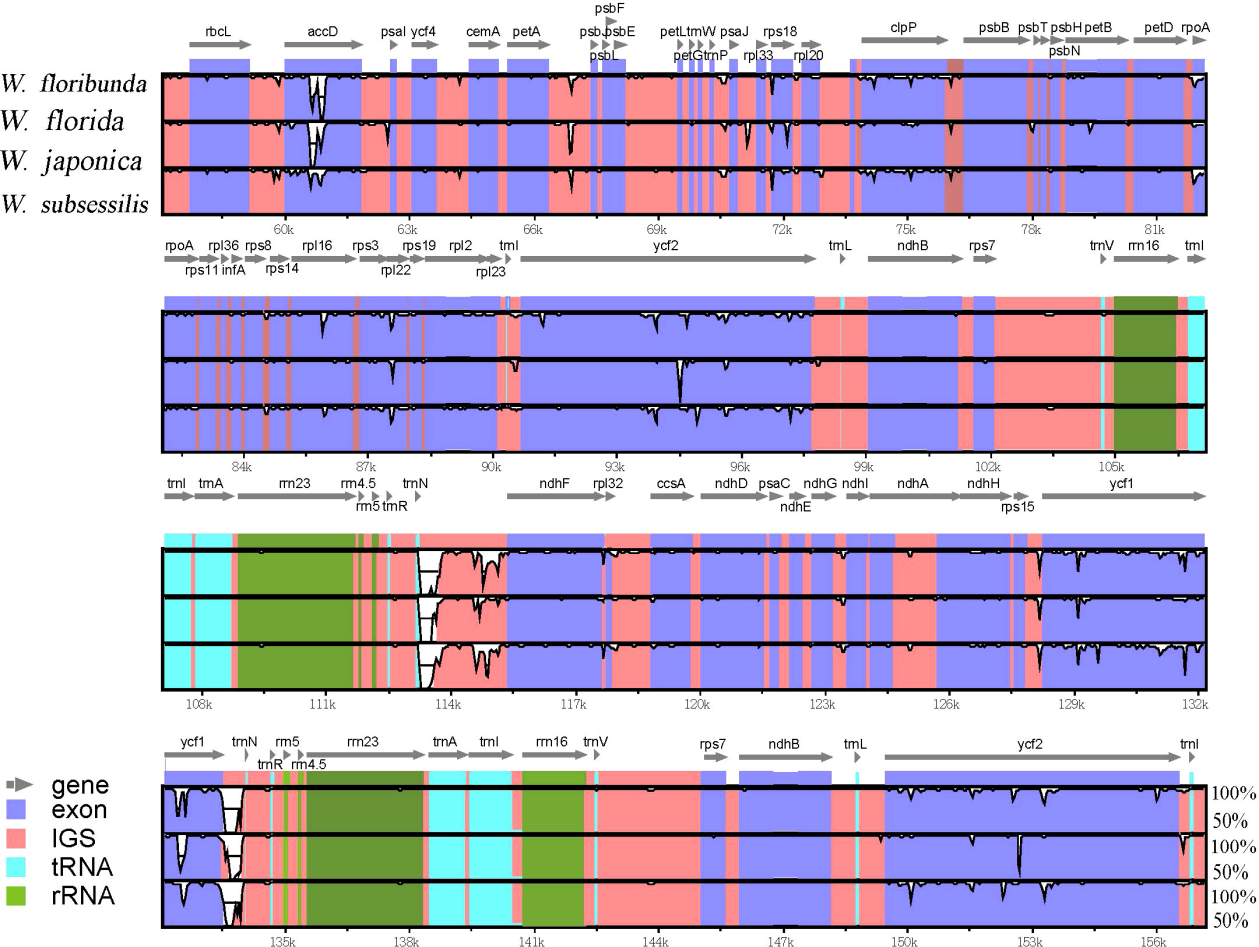
Sequence similarity analyses across the four *Weigela* plastomes generated through mVISTA. The vertical axis denotes the percentage of sequence identity, ranging from 50% to 100%. The horizontal axis corresponds to the coordinates within the chloroplast genome. Genome regions are color-coded to indicate protein-coding (exon), tRNAs or rRNAs, and intergenic regions. Genes are depicted by gray arrows.

To reveal the differences between the four *Weigela* species, we conducted a comprehensive analysis of the plastome sequences (**Figure 5**). We found that these plastomes of *Weigela* were highly similar, and no rearrangement had occurred. However, we detected some differences within the intergenic and intragenic regions among the plastomes in *Weigela*, especially in the LSC regions and the SSC regions (**Figure 5**). In view of this, there are a number of intergenic and intragenic areas for which DNA barcodes could be developed to differentiate *Weigela* species.

**Figure 6:**
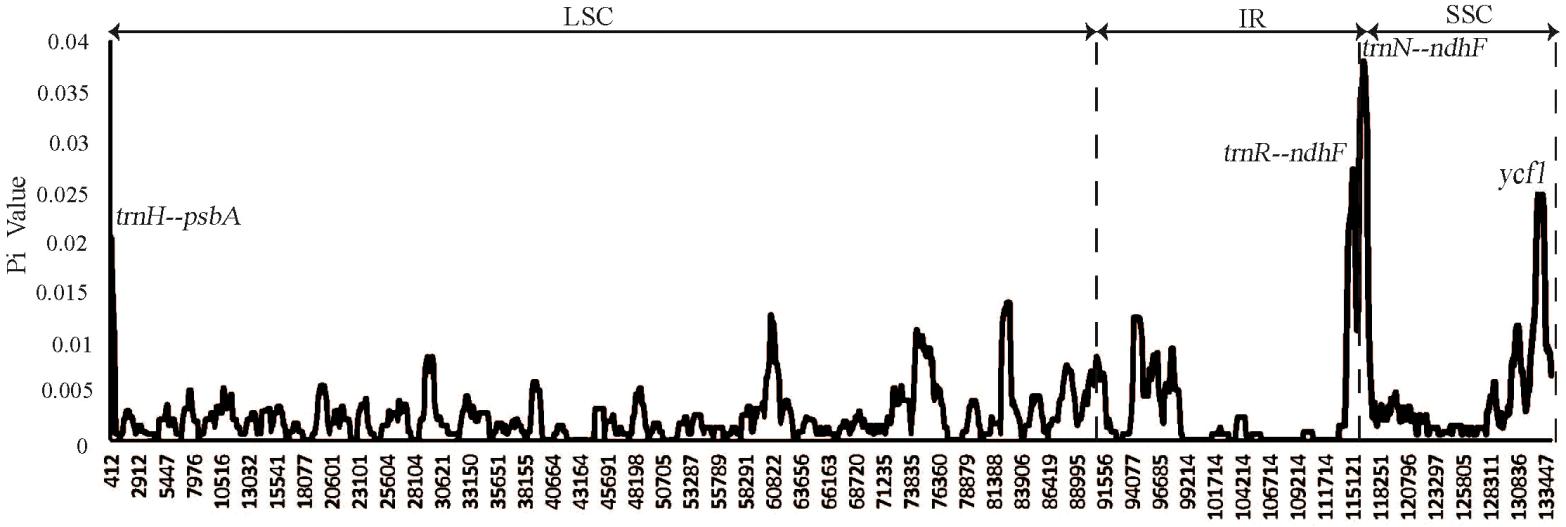
Nucleotide diversity (Pi) of plastomes among the four *Weigela* species. Four mutation hotspot areas (Pi > 0.002) were annotated. Pi values were calculated using an 800 bp sliding windows with 100 bp steps.

To facilitate a deeper analysis of the mutational hotspots within the plastomes of *Weigela*, DnaSP v6.12.03 was employed to analyze the nucleotide diversity (Pi) across the alignment of the four plastomes (**Figure 6**, **Table 3**). The observed Pi values ranged from 0 to 0.12333. At the threshold of Pi > 0.02, three mutational hotspots (*trnH-psbA*, *trnR-ndhF*, and *trnN-ndhF*) were suitable as candidate barcodes for *Weigela* species. The analysis of the nucleotide diversity in protein-coding genes showed that at the threshold of Pi > 0.02, only *ycf1* exhibited high nucleotide diversity and was deemed suitable for phylogenetic analysis.

**Table 3:** Four hypervariable regions of *Weigala*.

NO.	Region	Length(bp)	Variable sites	Parsimony informative sites	Nucleotide diversity (Pi)
1	trnH---psbA	812	29	14	0.02021
2	trnR---ndhF	2,307	71	14	0.02708
3	trnN--ndhF	2,130	107	20	0.03792
4	ycf1	1,487	45	11	0.02458

### Phylogenetic inference of the family Caprifoliaceae

**Figure 7:**
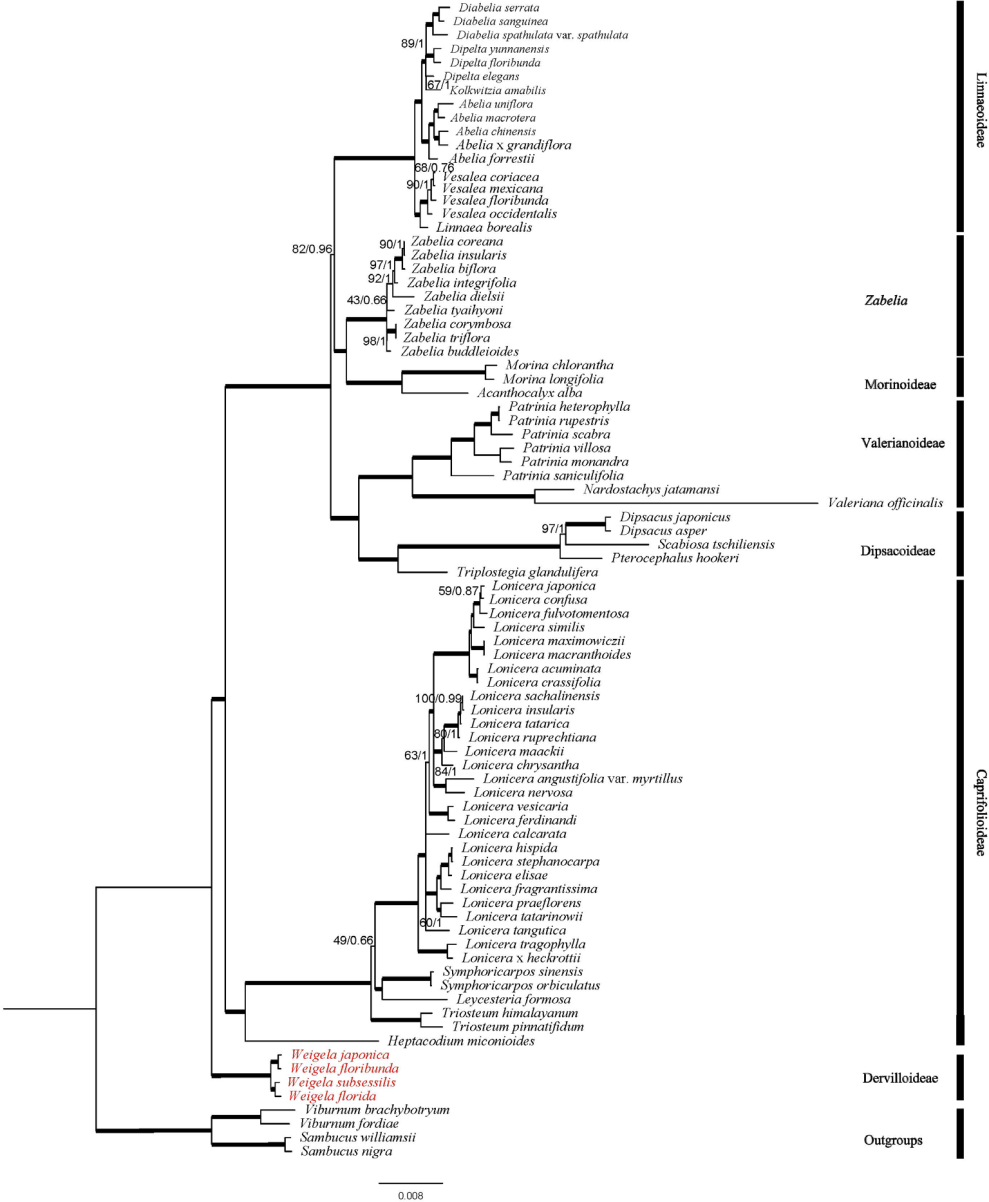
The phylogeny constructed by ML and BI methods based on 84 plastome genes. The numbers above the branches represent ML bootstrap support (BS) values and Bayesian posterior probabilities (PP) (100 BS/1.0 PP). Roots are thickened to indicate BS = 100 and PP = 1.

The phylogenetic topologies of the Caprifoliaceae inferred through ML and BI methods based on 83 gene sequences were consistent (**Figure 7**). All species of Caprifoliaceae constituted a strongly supported monophyletic group. In addition, the Caprifoliaceae family could be divided into seven clades: Diervilloideae, Caprifolioideae, Dipsacoideae, Valerianoideae, Morinoideae, *Zabelia*, and Linnaeoideae. Diervilloideae was located at the base of the whole family, and was retrieved as sister to the rest of the members with robust support (bootstrap (BS) value = 100% for the ML tree and posterior probability (PP) = 1.00 for the BI tree). Caprifolioideae was retrieved as sister to the other five clades with strong support. Moreover, *Zabelia* and Morinoideae formed a clade and were sister to Linnaeoideae. Dipsacoideae was sister to Valerianoideae, and the two were sister to Morinoideae + *Zabelia* + Linnaeoideae. The phylogeny within *Weigela* showed that *W. japonica* and *W. floribunda* were sister and *W. subsessilis* and *W. florida* were sister, with all *Weigela* species forming a monophyletic group.

## Discussion

In this study, we assembled four complete plastomes from *W. japonica*, *W. floribunda, W. subsessilis*, *W. florida*, and compared and analyzed the structural characteristics of these genomes. The four plastomes exhibited a high degree of conservation, with a typical quadripartite structure comprising one LSC, one SSC, and two IR regions. This result was consistent with the previous reports on plastomes of Caprifoliaceae (Fan et al., 2018; Peng et al., 2019; Park et al., 2021). The plastome size of *Weigela* species was 157,790~157,870 bp, which is not much different from other species of Caprifoliaceae (Fan et al., 2018; Peng et al., 2019).

The contractions or expansions at the boundaries of the IR regions are widely recognized as the primary processes leading to genome size variation (Chen et al., 2023; Zhang et al., 2022; Jia et al., 2024). Our results showed that the IR region of *W. subsessilis* was much shorter than that of the other three species, indicating that the expansion of IR regions caused a duplication of *ycf1* in the plastomes of *W. japonica*, *W. florida*, and *W. floribunda*. There were two types of SSC/IR boundary in the four species (**Figure 4**). *W. subsessilis* had a genomic structure in which *ycf1* was located only in the SSC region, and 83 bp were identified between the *ycf1* and IRa boundary in this species. The other three species had a different genomic structure, with *ycf1* located at the boundaries of the SSC and IRa regions. We also evaluated the variation in IRa and LSC junction regions, finding that the distributions and locations of genes within these regions displayed a high degree of variability. In consequence, changes in the SC/IR boundary may be the primary catalyst for the plastome size variation within *Weigela* species, especially in the IR region.

In a similar manner to the plastomes commonly found in angiosperms (Wicke et al., 2011; Daniell et al., 2016; Zhao et al., 2021), the plastomes of *Weigela* species exhibited a high similarity with other members of Caprifoliaceae (Yao et al., 2019; Wang et al., 2020). Nevertheless, some regions within these plastomes displayed a notable level of sequence variation. The mVISTA results showed that the sequence divergence in the IR region was lower compared to the LSC and the SSC regions (**Figure 5**). This result was attributed to the correction of sequence during gene replication and transcription of the two copies (Luo et al., 2021). Another reason contributing to the conservation of the IR regions is their crucial role in ensuring the structural stability of the plastome (Xiong et al., 2021; Wen et al., 2021; Park et al., 2021). From the results of the mVISTA analysis, we identified notable sequence variations in several intergenic regions and genes, including *trnH-psbA*, *trnR-ndhF*, *trnN-ndhF*, and *ycf1* (**Figure 6**). These regions with high sequence divergence have previously been reported in some other closely related lineages within Caprifoliaceae. For instance, Fan et al. (2018). compared eight plastomes of Caprifoliaceae species and identified 23 variant hotspot regions (containing genes and intergenic spacers) as candidate DNA barcodes that might be used as markers for phylogenetic analyses and species identification within the family Caprifoliaceae. These variant hotspot regions are potentially useful for phylogenetic analyses and interspecific divergence in the Caprifoliaceae. Our new findings also support the LSC region having higher sequence divergence than the IR and SSC regions (Wang et al., 2022a; Dong et al., 2022).

Repeat sequences play a pivotal role in the genomic rearrangement and structural stabilization of plastomes (Deng et al., 2018; Chen et al., 2023; Skuza et al., 2022). Such repeats are also important in understanding phylogenetic and biogeographic relationships, as well as population genetic characteristics, among species (Chen et al., 2023). In this study, a total of 1,263 long repeats (30-190 bp) without reverse and complementary repeats, and a total of 352 SSRs, were identified from the four *Weigela* plastomes (**Figure 2**). Among these long repeats, the number of tandem repeats was the highest, and the number of palindromic repeats was the lowest. In particular, most long repeat sequences were mainly located in noncoding areas, and a few long repeat sequences were found in shared genes. These repeats exhibited patterns similar to those previously reported, which are crucial components in the evolutionary dynamics of these plastomes (Sun et al., 2022; Wei et al., 2021).

Owing to their characteristics of abundance, maternal inheritance, and haploid nature, plastome SSRs (cpSSRs) have mainly been used for analysis of population genetic variation and gene flow (Xue et al., 2021; Ebert and Peakall, 2009), and are usually considered to be informative markers. The application and significance of cpSSR markers in other angiosperms have been frequently reported (Huang et al., 2018). In addition, we analyzed the numbers, types, and distribution of the cpSSRs in four *Weigela* plastomes. *W. floribunda* exhibited the highest count of cpSSRs (69), whereas *W. subsessilis* had the fewest (20). Consistent with the most recent results (Wang et al., 2022a; Zhou et al., 2018), we also determined that the mononucleotide-type SSRs is predominant in the plastome, with a bias toward A/T nucleotides, which corresponds with an A/T-rich plastome.

In recent years, studies increasingly demonstrate the unique advantages of plastome sequences in inferring phylogenetic relationships across various taxonomic levels (Guo et al., 2021; Xu and Wang, 2020). Based on the complete plastome sequences, researchers have successfully resolved numerous phylogenetic questions at deep-node levels of taxonomy, for example, in identifying the phylogenetic relationships among different angiosperms (Xiong et al., 2023) or among seven species of Papaveraceae (Wang et al., 2022a). This approach could lead to a better understanding of some complex evolutionary relationships in plant lineages. Meanwhile, the use of fewer plastome genes in analyses can only resolve shallow phylogenetic problems to a certain extent. Previously, Li et al. (1999). reconstructed the internal phylogenetic relationships of Hamamelidaceae through the chloroplast gene *matK*. The results indicated that the plastome gene resolved the phylogenetic relationships among subfamilies/families within Hamamelidaceae well, but failed to resolve the phylogeny at the species level. In this study, we constructed the phylogeny of the Caprifoliaceae based on whole plastomes. The results showed that *Weigela* belongs to Diervilloideae, which is an early diverging lineage in the family Caprifoliaceae, and was retrieved as sister to the rest of the members with strong support. *Zabelia* and Morinoideae formed a clade and were sister to Linnaeoideae. Dipsacoideae was sister to Valerianoideae, and the two were retrieved as sister groups to Morinoideae + *Zabelia* + Linnaeoideae. This is consistent with the results inferred from transcriptomes and plastomes (Li et al., 2024). Furthermore, this phylogeny also confirmed the phylogenetic relationships of the four studied species in *Weigela*, and the support value of each clade was significantly higher than the previous phylogenies based on ITS sequences and cpDNA regions (Kim and Kim, 1999; Meeler, 2018). In addition, the short branch length of the four *Weigela* species indicated that species distributed in the northeast Asia might have undergone a rapid radiation (Chang, 1997).

## Conclusions

In conclusion, we newly sequenced and assembled four complete plastomes of *Weigela*, and conducted comparative analysis between them. On this basis, we also reconstructed the phylogeny of the whole Caprifoliaceae family with the help of 75 published plastomes from other Caprifoliaceae genera. Our comparative plastomes analysis has shown that the plastomes of *Weigela* are highly conserved. Moreover, only a few regions of high sequence divergence were detected throughout the whole plastome. The results of the phylogenetic analysis provide a stable phylogenetic framework for Caprifoliaceae and the phylogenetic position of *Weigela*. Furthermore, the phylogenetic relationships of the four species of *Weigela* were confirmed.

## Data Availability

The datasets presented in this study can be found in online repositories. The names of the repository/repositories and accession number(s) can be found in the article/**Supplementary Material**.

## Supplementary material

Supplementary Figure S1 Gene map of four *Weigela* species plastome. Genes located outside the circle are transcribed counterclockwise, while genes inside the circle are transcribed clockwise. The dark gray area and light gray area of the inner circle represent the ratio of GC content to AT content in the genome, respectively. Different colored blocks represent genes from different functional groups.
