# Supplementary figures and images for: Comparative plastomes sheds light on phylogeny of Weigela
Source: Front Plant Sci. 2024 Oct 29;15:1487725. doi: 10.3389/fpls.2024.1487725 (PMC11554533; doi:10.3389/fpls.2024.1487725)

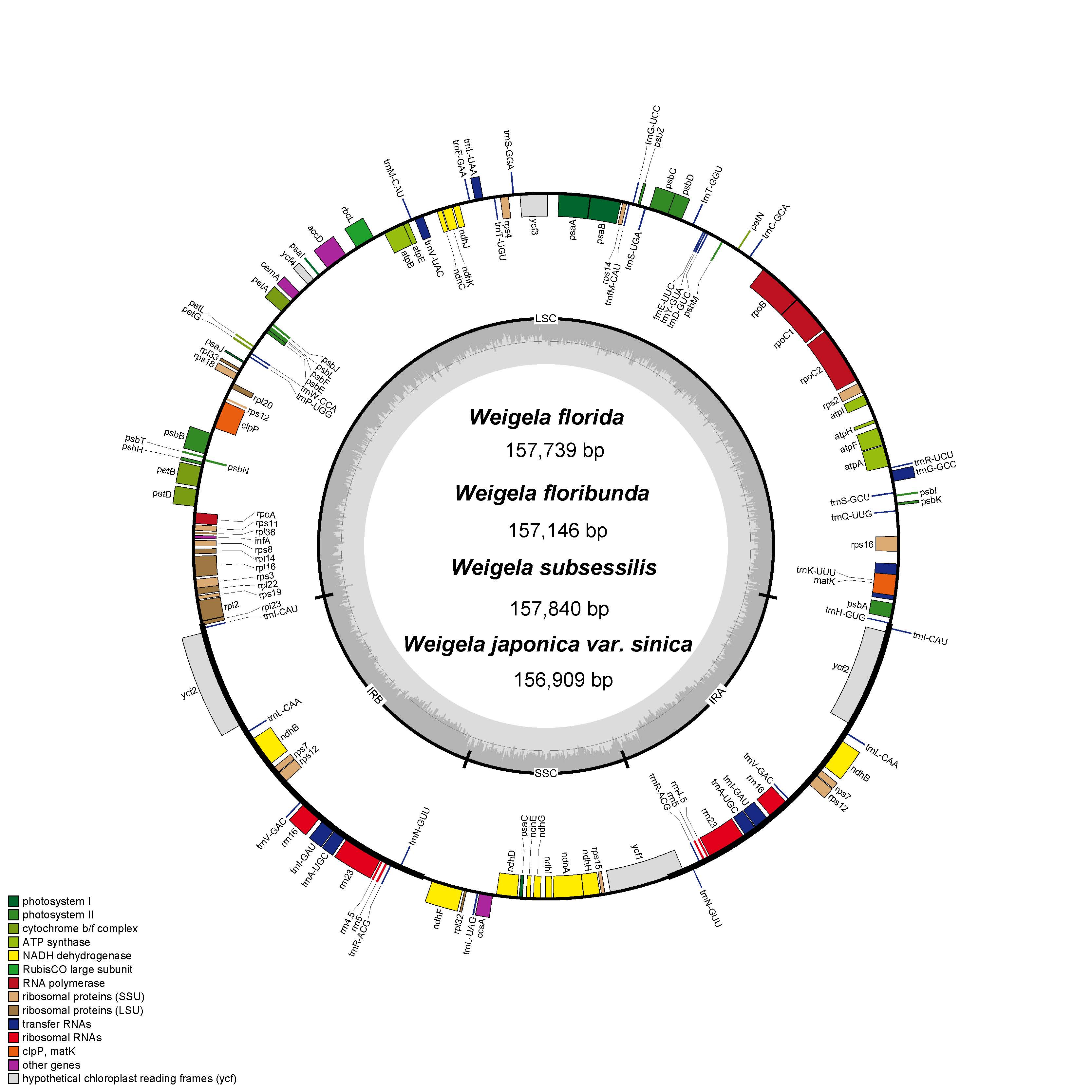

Supplement: Supplementary Figure S1 — Gene map of four Weigela species plastome. Genes located outside the circle are transcribed counterclockwise, while genes inside the circle are transcribed clockwise. The dark gray area and light gray area of the inner circle represent the ratio of GC content to AT content in the genome, respectively. Different colored blocks represent genes from different functional groups. [file DataSheet1.zip › Figure S1.jpg]
